# Factors Associated with Alcohol Consumption in Hepatitis B Carriers: A Nationwide Study in the Republic of Korea

**DOI:** 10.1371/journal.pone.0110144

**Published:** 2014-11-11

**Authors:** Boyoung Park, Kyu-Won Jung, Chang-Mo Oh, Kui Son Choi, Mina Suh, Jae Kwan Jun

**Affiliations:** National Cancer Control Institute, National Cancer Center, Goyang-si, Korea; Yonsei University College of Medicine, Korea, Republic Of

## Abstract

This study was conducted to investigate the prevalence of alcohol consumption and identify the sociodemographic factors associated with alcohol consumption among individuals with hepatitis B virus(HBV) infection. We used data from the Korean National Health and Nutrition Examination Surveys, a nationwide survey conducted between 2007 and 2011. “Monthly alcohol consumption” was defined as having consumed alcohol at least once per month during the past year, and “high-risk alcohol consumption” was defined as having consumed alcohol twice or more per week and, for males, having consumed at least 60 g of alcohol on one occasion or, for females, having consumed at least 40 g of alcohol on more than one occasion. The prevalence of monthly alcohol consumption was 53.2%, and that of high-risk alcohol consumption was 11.8% among HBV carriers. Less education was associated with both monthly and high-risk alcohol consumption(OR = 1.75 [95% CI = 1.02−3.02] for monthly alcohol consumption among those with less than a high school education; OR = 2.48 [95% CI = 1.19−5.17] for high-risk alcohol consumption among those with less than a high school education and OR = 2.02 [95% CI = 1.12−3.64] among those with a high school education). Additionally, smoking and being male increased the risk of alcohol consumption, and older age and having a normal body mass index decreased the risk. HBV carriers who were less educated, overweight, and smokers were more likely to consume alcohol or meet criteria for high-risk drinking. Health policies and intervention programs aimed at promoting a generally healthy lifestyle in HBV carriers should consider educational inequalities and alcohol consumption.

## Introduction

Hepatitis B virus (HBV) infection is a major global public health issue. It is estimated that, worldwide, more than 2 billion people are infected with HBV and that 378 million are chronic carriers. Additionally, about 4.5 million new HBV infections develop worldwide each year; about one-quarter of these progress to further liver disease and approximately 600,000 individuals die of HBV-related causes each year [1]. The disease burden of HBV infection is especially high in Asian countries. Indeed, about 75% of chronic HBV carriers reside in the Asia–Pacific region, and 15–25% die of HBV-related liver diseases [2]. People who are chronically infected with HBV are at high risk of developing life-threatening chronic diseases, such as liver cirrhosis and hepatocellular carcinoma (HCC) [3]. HCC accounts for 70–85% of primary liver cancer cases [4], which is the sixth most common cancer and the third leading cause of death from cancer worldwide [5].

The outcome of HBV infection is influenced by several viral and host factors, and alcohol consumption is one of the host factors that affects the progress of HBV infection [6]. Alcohol consumption itself is also a major cause of chronic liver diseases, such as liver cirrhosis and HCC, and it contributes to the progression of liver damage when other risk factors exist [7]. Alcohol intake independently increases the risk of liver cirrhosis and HCC in HBV carriers [8]. Some researchers have even suggested a multiplicative interactive effect between HBV infection and heavy alcohol consumption on the risk of liver cirrhosis [7]. In terms of HCC development, compared with non-viral-induced cirrhosis, cirrhosis related to viral infection, especially chronic HBV infection, is associated with a higher risk of developing HCC [9]. Several previous studies have shown that alcohol consumption and HBV infection operate synergistically [10]–[12] and share mechanisms of action in the development of HCC [13].

The Republic of Korea is among the regions with a high prevalence (≥8%) of HBV infection [14]. Although the prevalence of HBV infection has decreased because of the nationwide vaccination program implemented in 1995, HBV infection is still considered endemic in the Republic of Korea, which is now classified as intermediate in this regard [15].

The prevalence of drinkers and the amount of alcohol consumed per capita in the Republic of Korea is among the highest worldwide and the prevalence of alcohol consumption in the population has been increasing [16]. Considering the disease burden of HBV infection, efforts to combat the development of liver disease must focus not only on the primary prevention of HBV infection through HBV vaccination but also on the modification of risk factors, such as reducing alcohol consumption in HBV carriers.

Thus, in this study, we investigated the prevalence of alcohol consumption, a major factor that operates synergistically with other factors in the development of liver disease, among HBV carriers and compared it with that among HBV non-carriers. We also identified the sociodemographic factors associated with alcohol consumption in individuals with HBV infection using a nationally representative sample of the population of the Republic of Korea.

## Materials and Methods

### Study population

This study used data from the Korean National Health and Nutrition Examination Surveys (KNHANES), a series of cross-sectional, population-based nationally representative surveys that relied on independent rolling survey sampling based on a complex survey design. This survey had been implemented every 3 years between 1998 and 2005; since 2007, it has been conducted annually. KNHANES uses a standardized questionnaire to examine health behavior, a health examination survey, and a nutrition survey using a food-frequency questionnaire. Details of the survey have been described fully on the KNHANES official website, and the survey data are publicly available (http://knhanes.cdc.go.kr/). The study protocol was approved by the Institutional Review Board of the Korean Centers for Disease Control and Prevention.

Our analysis included data from the KNHANES conducted between 2007 and 2011. During the period, 53,232 individuals were sampled, and 42,347 participated in the survey (response rate: 71.2% in 2007, 77.8% in 2008, 82.8% in 2009, 81.9% in 2010, and 80.4% in 2011). Individual HBV surface antigen (HBsAg) values were measured in serum with an electrochemiluminescence immunoassay (Roche Diagnostics, Switzerland) in those aged 10 years or older. Of these, we selected 28,340 individuals aged 20 years or older with complete HBsAg test results; 27,318 of this group were negative for HBsAg, and 1,022 were positive. Thus, 3.7% of the population aged 20 years or older was infected with HBV, and the weighted prevalence was 3.5%. We defined those whose serum was HBsAg-positive as HBV carriers and those whose serum was HBsAg-negative as HBV non-carriers.

### Data collection

Participants' drinking patterns, including alcohol consumption, frequency of alcohol consumption during the previous year, and amount of alcohol consumed on one occasion, were accessed by questionnaire. The World Health Organization (WHO) defines high-risk drinking as drinking 60 g of alcohol or more for males and 40 g of alcohol or more for females on a single day [17]. We defined “monthly alcohol consumption” as having consumed alcohol once or more per month during the past year, and “high-risk alcohol consumption” as having consumed alcohol twice or more per week and, for males, having consumed at least 60 g of alcohol on one occasion or, for females, having consumed at least 40 g of alcohol on one occasion [18].

Demographic data (sex, age, area of residence), socioeconomic factors (monthly household income, educational level), life style-related factors (subjective health status, recognition of HBV infection status, smoking) were collected using a standardized questionnaire. We defined “recognition of HBV infection” as being serum HBsAg-positive and diagnosed with HBV infection by a physician. Body mass index (BMI) was calculated based on the height and weight data collected during the health examination.

### Statistical analyses

All analyses incorporated sampling weights, stratification, and clustering. The total proportions of baseline characteristics among HBV carriers and non-carriers were compared with the *chi*-square test. In addition, the annual prevalence of the monthly alcohol consumption and high-risk alcohol consumption for 2007 and 2011 was analyzed. We performed multiple logistic regression to estimate the odds ratios (ORs) and 95% confidence intervals (CIs) for the association between sociodemographic factors and monthly alcohol consumption and high-risk alcohol consumption among HBV carriers to control for the effects of other sociodemographic variables and showed the frequency and percent of each sociodemographic factor. A *p*-value of <0.05 was considered to indicate statistical significance. All statistical analyses were performed using the SAS software (ver. 9.1; SAS, Inc., Cary, NC, USA).

## Results

**Figure 1:**
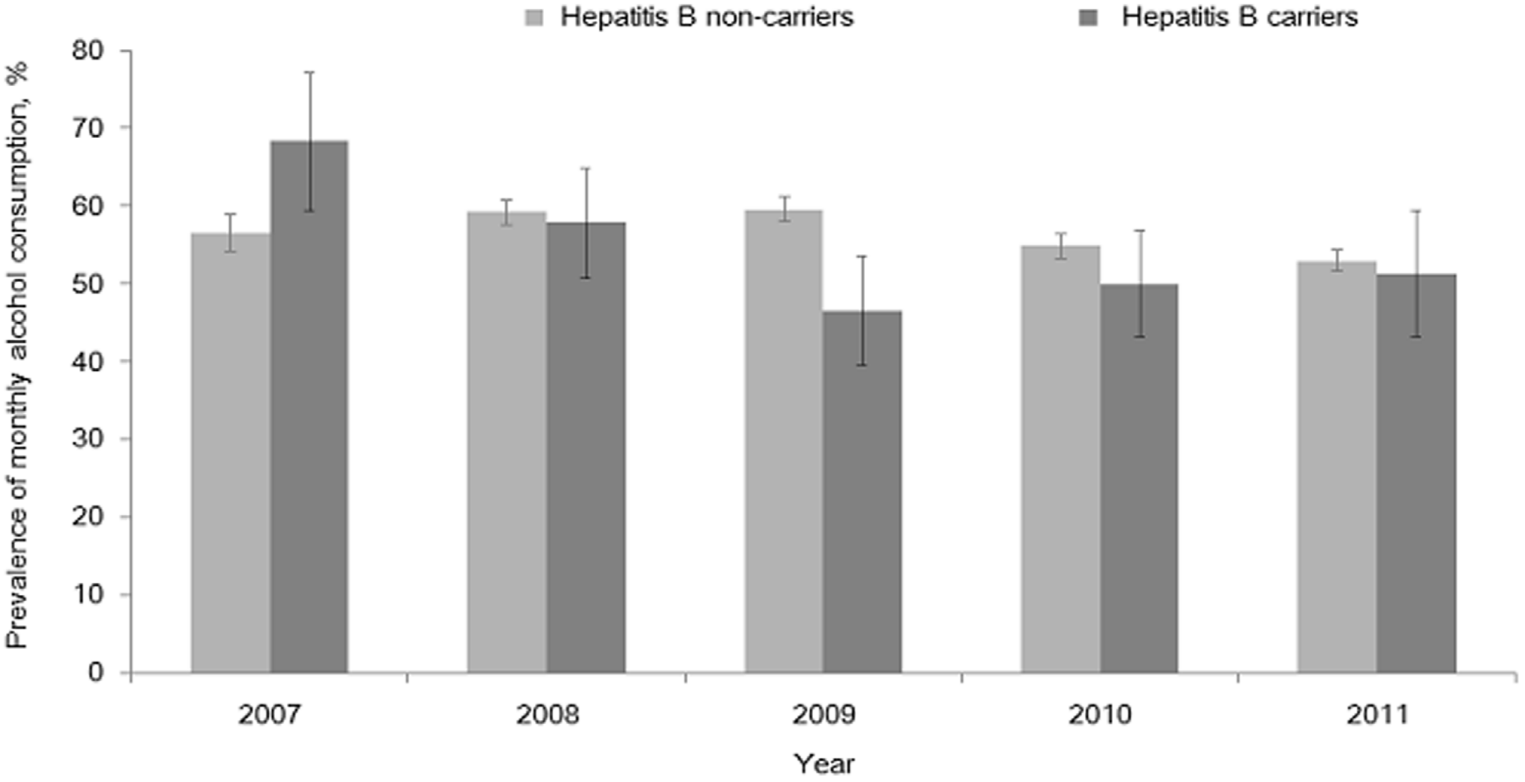
The annual prevalence of monthly alcohol consumption and high-risk alcohol consumption during the year of 2007–2011 among HBV carriers and non-carriers aged 20 or older.

**Figure 2:**
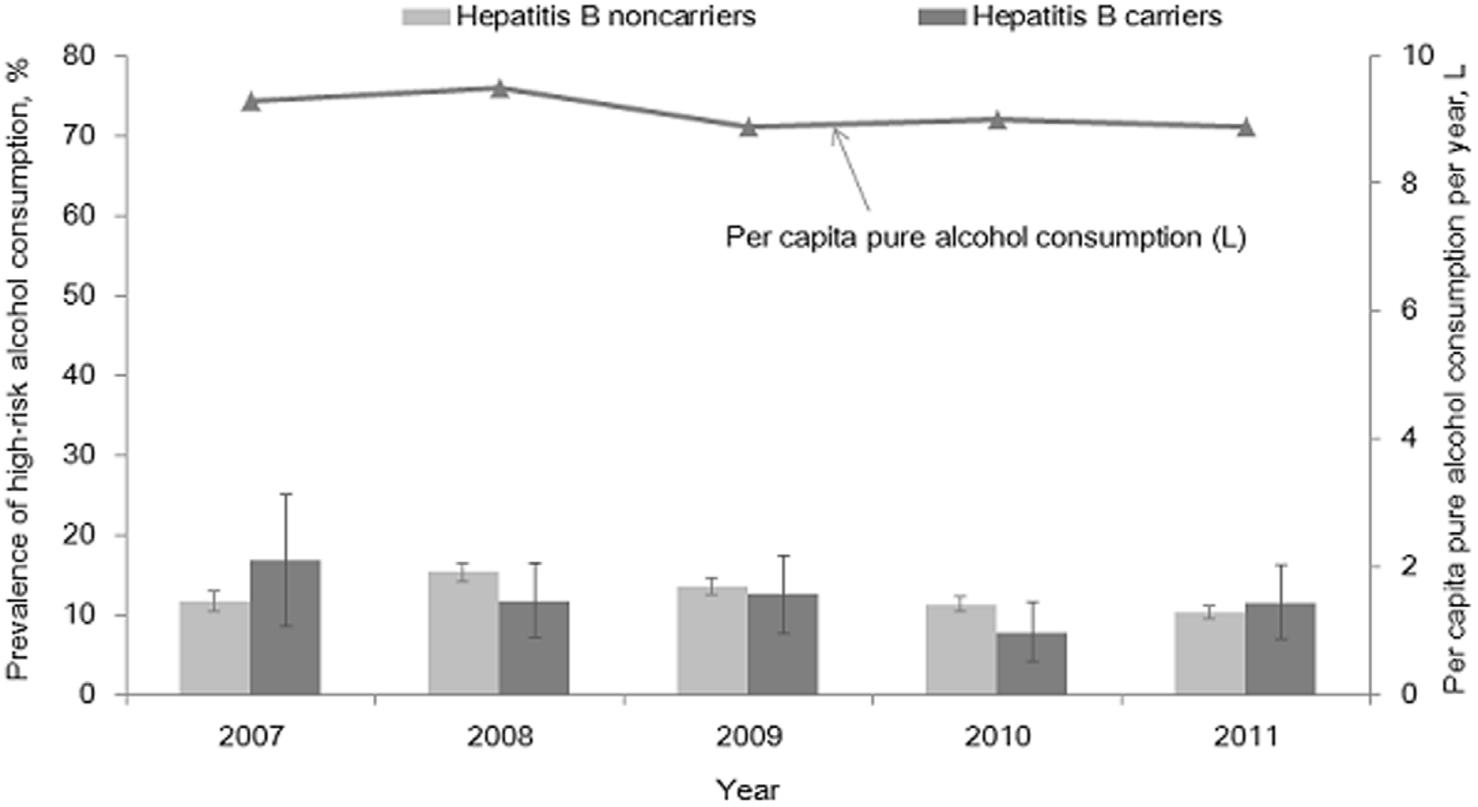
Trends in annual prevalence of high-risk alcohol consumption during the year of 2007–2011 among hepatitis B carriers and non-carriers aged 20 or older and per capita pure alcohol consumption per year in general population. The prevalence of high-risk alcohol consumption was calculated from Korean National Health and Nutrition Examination Surveys and Korean Statistical Information Service data was applied for per capita pure alcohol consumption.

Among HBV carriers, 53.2% consumed alcohol at least once per month during the previous year, but this figure did not differ significantly from that for non-carriers (56.0% for non-carriers, *P* = 0.168). The prevalence of high-risk alcohol consumption was 11.8% in HBV carriers and 12.0% in HBV non-carriers. The prevalence of high-risk alcohol consumption also did not significantly differ between HBV carriers and non-carriers (*P* = 0.867). Figure 1 and 2 shows the annual prevalence of the monthly alcohol consumption and high-risk alcohol consumption for the years 2007 and 2011 among HBV carriers and non-carriers age 20 years or older.

Table 1 compares the baseline characteristics of HBV carriers and non-carriers in 2007–2011 in the Republic of Korea. Among HBV carriers, 52.4% were males and 47.6% were females. Most HBV carriers (79.1%) in Korea did not recognize their infection status, nearly 50% of HBV carriers considered their health status to be moderate, and 44% of HBV carriers were smokers. Compared with non-carriers, more HBV carriers tended to be male, in their 40s and 50s, and to consider their health status bad or moderate. Other baseline characteristics were not significantly different.

**Table 1:** Weighted prevalence of baseline characteristics of participants aged 20 years and older with hepatitis B virus surface antigen and non-carriers (KNHANES 2007–2011).

	Hepatitis B carriers			Hepatitis B non-carriers			P-value
	Sample size	Prevalence (%)	95% CI	Sample size	Prevalence (%)	95% CI	
Overall	1022			27318			
Sex							
Male	492	52.4	(48.9–56.0)	11676	46.0	(45.5–46.6)	0.001
Female	530	47.6	(44.0–51.1)	15642	54.0	(53.4–54.5)	
Age							
<40	300	31.2	(18.5–24.4)	8748	35.6	(34.4–36.8)	<0.001
40–49	252	25.1	(19.1–25.4)	5298	20.3	(19.5–21.1)	
50–59	224	22.7	(21.8–28.4)	4935	18.3	(17.5–19.0)	
≥60	246	21.4	(27.8–34.6)	8337	25.9	(24.8–27.0)	
Area of residence							
Urban	768	79.3	(75.4–83.2)	20719	80.1	(77.5–82.8)	0.610
Rural	254	20.7	(16.8–24.6)	6599	19.9	(17.2–22.5)	
Household income							
Low	166	14.3	(11.8–16.9)	5446	17.5	(16.5–18.4)	0.161
Mid–low	264	26.3	(23.0–29.6)	6757	25.0	(23.9–26.0)	
Mid–high	260	26.9	(23.3–30.6)	7317	27.8	(26.8–28.9)	
High	310	30.5	(26.9–34.0)	7243	27.8	(26.4–29.2)	
Missing	22	1.9	(1.0–2.9)	555	1.9	(1.6–2.2)	
Education							
Less than high school	376	33.3	(29.7–36.8)	10378	33.1	(31.8–34.3)	0.319
High school	328	32.7	(29.1–36.3)	9067	35.6	(34.6–36.5)	
College or more	306	32.0	(28.2–35.8)	7543	29.9	(28.7–31.0)	
Missing	12	2.1	(0.9–3.3)	330	1.5	(1.3–1.8)	
Body mass index							0.170
<18.5	31	3.2	(1.9–4.4)	1221	4.6	(4.3–5.0)	
18.5–24.9	640	61.8	(58.4–65.2)	17312	63.1	(62.4–63.8)	
≥25.0	347	34.5	(31.1–37.8)	8659	31.8	(31.1–32.5)	
Missing	4	0.5	(−0.1–1.1)	126	0.4	(0.3–0.6)	
Subjective health status							
Bad	246	20.9	(17.9–23.8)	5975	19.2	(18.6–19.9)	<0.001
Moderate	469	49.6	(45.9–53.4)	10785	42.3	(41.4–43.2)	
Good	295	27.4	(24.1–30.7)	10243	37.0	(36.1–37.9)	
Missing	12	2.1	(0.9–3.3)	315	1.5	(1.2–1.7)	
Smoking							
Smoker	414	43.7	(40.1–47.3)	10624	41.0	(40.4–41.7)	0.278
Non–smoker	597	54.5	(50.9–58.1)	16384	57.5	(56.9–58.1)	
Missing	11	1.8	(0.7–2.9)	310	1.5	(1.2–1.7)	
Awareness of HBV infection							
Unaware	809	79.1	(76.0–82.2)	-			
Aware	213	20.9	(17.8–24.0)	-			

Table 2 presents the sociodemographic factors associated with monthly alcohol consumption in HBV carriers. Being male, smoking, and less education were significantly associated with higher rates of monthly alcohol consumption (OR = 2.56 [95% CI = 1.62−4.03, *P*<0.001] for males; OR = 2.28 [95% CI = 1.47−3.54, *P*<0.001] for smokers; and OR = 1.75 [95% CI = 1.02−3.02, *P* = 0.044] for those with less than a high school education vs. those with at least some college). People older than 50 drank less than did people younger than 40 (*P*<0.001), and low household income and normal BMI (18.5–24.9) were significantly associated with less monthly drinking (OR = 0.43 [95% CI = 0.24−0.80, *P* = 0.008] for the those in the first quartile of household income vs. those in the fourth quartile, and OR = 0.69 [95% CI = 0.48−0.99, *P* = 0.045] for normal BMI vs. those with BMI ≥25 m/Kg^2^). Area of residence, recognition of HBV infection, and subjective health status were not associated with monthly drinking.

**Table 2:** Multivariate analysis of association between monthly alcohol consumption^a^ and sociodemographic factors in HBV carriers.

	Monthly alcohol consumers		Non-consumers		Odds ratio	95% CI	*P-value*
	N	%^b^ (95% CI)	N	%^b^ (95% CI)			
Sex							
Male	327	68.1(63.8–72.4)	157	34.4(29.4–39.4)	2.56	(1.62–4.03)	<0.01
Female	175	31.9(27.6–36.2)	349	65.6(60.6–70.6)	1		
Age							
<40	179	36.9(32.3–41.4)	166	29.9(25.3–34.5)	1		
40–49	143	29.6(24.8–34.3)	117	24.8(20.1–29.6)	0.68	(0.43–1.06)	0.09
50–59	104	20.0(15.9–24.1)	108	20.8(16.7–24.8)	0.31	(0.19–0.50)	<0.01
≥60	76	13.5(10.2–16.9)	115	24.5(20.3–28.6)	0.23	(0.13–0.42)	<0.01
Area of residence							
Urban	383	79.3(74.7–83.8)	373	79.3(74.8–83.9)	0.76	(0.50–1.18)	0.22
Rural	119	20.7(16.2–25.3)	133	20.7(16.1–25.2)	1		
Household income							
Low	55	8.6(6.0–11.1)	108	20.6(16.3–24.8)	0.43	(0.24–0.80)	0.01
Mid–low	138	28.7(24.0–33.5)	124	24.2(19.6–28.7)	1.02	(0.66–1.59)	0.93
Mid–high	133	27.4(22.6–32.3)	123	26.5(21.7–31.4)	0.65	(0.42–1.01)	0.06
High	167	33.9(29.2–38.6)	141	27.1(22.7–31.6)	1		
Education							
Less than high school	148	27.3(23.0–31.6)	228	41.6(36.3–46.8)	1.75	(1.02–3.02)	0.04
High school	184	36.0(30.9–41.2)	142	29.8(25.0–34.6)	1.34	(0.88–2.05)	0.18
College or more	169	36.3(31.3–41.3)	135	28.3(23.6–33.0)	1		
Body mass index							
<18.5	15	3.4(1.6–5.2)	15	2.9(1.2–4.7)	1.12	(0.49–2.58)	0.79
18.5–24.9	302	58.2(53.3–63.1)	326	64.8(59.8–69.7)	0.69	(0.48–0.99)	0.04
≥25.0	182	37.5(32.6–42.3)	164	32.3(27.6–37.0)	1		
Awareness of HBV infection							
Unaware	394	77.1(72.4–81.7)	401	80.4(76.3–84.5)	1.02	(0.67–1.55)	0.92
Aware	108	22.9(18.3–27.6)	105	19.6(15.5–23.7)	1		
Subjective health status							
Bad	94	17.5(13.8–21.1)	151	25.6(21.0–30.2)	0.75	(0.48–1.18)	0.21
Moderate	249	53.3(48.4–58.1)	219	47.6(42.4–52.9)	0.96	(0.66–1.39)	0.81
Good	158	28.9(24.4–33.4)	135	26.4(21.8–31.0)	1		
Smoking							
Smoker	284	58.7(54.0–63.4)	130	28.6(23.8–33.5)	2.28	(1.47–3.54)	<0.01
Non–smoker	218	41.3(36.6–46.0)	375	71.2(66.4–76.0)	1		

a “Monthly alcohol consumption” was defined as having consumed alcohol at least once per month during the past year.

b The sum of the percentage may not be 100 because the number of missing values were not presented. Missing values were treated as dummy variables in the analysis.

Table 3 shows the associations between individual sociodemographic factors and high–risk alcohol consumption in HBV carriers. Similar to the association with monthly alcohol consumption, being male, smoking, and less education were significantly associated with a higher prevalence of high–risk alcohol consumption (OR = 3.46 [95% CI = 1.33−9.00, *P*<0.011] for males; OR = 4.15 [95% CI = 1.75−9.85, *P* = 0.001] for smokers; and OR = 2.48 [95% CI = 1.19−5.17, *P* = 0.016] for those with less than high school education and OR = 2.02 [95% CI = 1.12−3.64, *P* = 0.020] for those with a high school education vs. those with at least some college). Being older, having a normal BMI, and moderate self-rated health status were associated with less high–risk alcohol consumption. Area of residence, household income, and recognition of HBV infection were unrelated to high–risk alcohol consumption.

**Table 3:** Multivariate analysis of association between high-risk alcohol consumption^a^ and sociodemographic factors in HBV carriers.

	High-risk alcohol consumers		Non-high-risk consumers		Odds ratio	95% CI	*P-value*
	N	%^b^ (95% CI)	N	%^b^ (95% CI)			
Sex							
Male	95	87.0(80.9–93.0)	397	47.8(44.1–51.6)	3.46	(1.33–9.00)	0.01
Female	12	13.0(7.0–19.1)	518	52.2(48.4–55.9)	1		
Age							
<40	9	8.0(3.2–12.8)	237	23.2(20.1–26.4)	1		
40–49	17	15.7(8.4–23.0)	207	23.1(19.7–26.6)	0.92	(0.50–1.70)	0.79
50–59	41	38.7(29.7–47.6)	211	23.3(20.0–26.6)	0.29	(0.13–0.64)	<0.01
≥60	40	37.6(28.0–47.2)	260	30.3(26.7–34.0)	0.19	(0.06–0.60)	<0.01
Area of residence							
Urban	78	81.5(75.4–87.6)	690	79.0(75.0–83.1)	1.09	(0.61–1.93)	0.78
Rural	29	18.5(12.4–24.6)	225	21.0(16.9–25.0)	1		
Household income							
Low	9	6.1(1.3–10.8)	157	15.4(12.7–18.2)	0.49	(0.17–1.42)	0.19
Mid–low	33	29.7(21.5–37.9)	231	25.8(22.4–29.3)	1.28	(0.67–2.44)	0.45
Mid–high	31	29.4(21.3–37.5)	229	26.6(22.9–30.3)	0.84	(0.44–1.63)	0.61
High	33	34.4(25.6–43.3)	277	29.9(26.3–33.6)	1		
Education							
Less than high school	26	23.4(15.7–31.1)	14	2.4(1.1–3.7)	2.48	(1.19–5.17)	0.01
High school	44	42.8(33.4–52.2)	395	45.7(41.6–49.7)	2.02	(1.12–3.64)	0.02
College or more	37	33.8(24.6–42.9)	506	51.9(47.9–55.9)	1		
Body mass index							
<18.5	3	4.2(1.5–7.0)	28	3.0(1.8–4.2)	1.23	(0.31–4.91)	0.77
18.5–24.9	55	49.1(40.3–57.8)	585	63.5(60.0–67.0)	0.51	(0.31–0.82)	0.01
≥25.0	49	46.7(38.0–55.4)	298	32.8(29.4–36.3)	1		
Awareness of HBV infection							
Unaware	85	76.4(67.6–85.2)	724	79.4(76.3–82.6)	1.16	(0.63–2.13)	0.64
Aware	22	23.6(14.8–32.4)	191	20.6(17.4–23.7)	1		
Subjective health status							
Bad	19	18.6(11.0–26.2)	227	21.2(18.0–24.3)	0.62	(0.32–1.22)	0.16
Moderate	47	44.1(35.2–53.1)	422	50.4(46.5–54.2)	0.49	(0.28–0.84)	0.01
Good	41	37.3(28.2–46.4)	254	26.1(22.8–29.4)	1		
Smoking							
Smoker	88	81.1(73.5–88.7)	326	38.7(34.9–42.5)	4.15	(1.75–9.85)	<0.01
Non–smoker	19	18.9(11.3–26.5)	578	59.3(55.5–63.0)	1		

a “High-risk alcohol consumption” was defined as having consumed alcohol twice or more per week and, for males, having consumed at least 60 g of alcohol on one occasion or, for females, having consumed at least 40 g of alcohol on more than one occasion.

b The sum of the percentage may not be 100 because the number of missing values were not presented. Missing values were treated as dummy variables in the analysis.

## Discussion

This study found that 53.2% of HBV carriers consumed alcohol on a monthly basis and that 11.8% of this group met criteria for high-risk alcohol consumption, but these figures did not significantly differ from those for HBV non-carriers. Less education, smoking, and being overweight were associated with monthly drinking and high-risk drinking in HBV carriers, and we confirmed the existence of educational inequalities in the alcohol consumption of HBV carriers. To our knowledge, this is the first published study to investigate the prevalence of alcohol consumption among HBV carriers who are at high risk for chronic liver disease.

The proportion of Korean adults who drink alcohol is among the highest in the world and is increasing [19]. The proportion of high-risk alcohol drinkers has also been increasing [18], and both the prevalence of high-risk alcohol consumption and total alcohol consumption per capita are high in the Republic of Korea compared with most other countries [16].

In the Republic of Korea, the prevalence of those with serum HBsAg-positive status has been decreasing rapidly, from 8.6% in 1980 [15] to 3.2% in 2009, due to a nationwide HBV vaccination program implemented in 1995 [20]. However, the decrease in the disease burden related to chronic liver diseases, including liver cancers, is not as dramatic as is the decrease in the prevalence of HBsAg. To further decrease the risk for developing liver disease, it has been recommended that HBV carriers stop drinking [6]. Indeed, the treatment guidelines for chronic hepatitis B developed by the Korean Association for the Study of the Liver recommend that HBV carriers avoid drinking [21]. However, more than 50% of HBV carriers drank alcohol and more than 10% were heavy drinkers. Thus, in addition to current efforts that focus on reducing high-risk groups, such as HBV carriers, through vaccination, more efforts are needed to prevent the gradual development of liver disease in general, especially in high-risk groups.

Educational attainment and alcohol use have been associated with each other in several previous studies. Several studies have suggested that *less* education is associated with alcohol dependence [22]–[24], whereas others have proposed that *more* education is associated with *increased* daily alcohol consumption or problematic drinking [25] or that there is *no* association between these factors [26]. In this study, we showed that lower educational levels were associated with increased monthly alcohol consumption and high-risk alcohol consumption in HBV carriers. The odds ratios of educational attainment were more significant for high-risk alcohol consumption than for monthly alcohol consumption, showing educational disparities in both alcohol consumption, in general, and heavy drinking patterns, in particular. In Korea, there are differences in mortality due to liver cancer and liver diseases according to educational attainment, and these differences reflect major inequalities [27]. Our results showing differences in drinking according to educational level in HBV carriers may, at least in part, explain the inequalities in liver cancer and liver disease mortality.

Our results showed that smokers who were HBV carriers were more likely to drink alcohol on a monthly basis and to be heavy drinkers, which is similar to previous results indicating that smoking and drinking are related in the general population [28]. Similar to previous studies that showed an association between obesity and alcohol consumption [29], [30], the odds that individuals who were of normal weight (18.5≤ BMI <25 kg/m^2^) consumed alcohol were significantly lower than were those that overweight or obese people (BMI ≥25 kg/m^2^) did so. Additionally, our results showed no statistically significant differences in monthly drinking or high-risk drinking between obese people (BMI ≥25 kg/m^2^) and people with BMI <18.5 kg/m^2^. Considering the synergistic effects of smoking and HBV infection and of obesity and HBV infection on the risk of HCC [12], [29], the higher prevalence of smokers or overweight/obese people with HBV infection may further increase the risk of developing HCC.

To reduce the disease burden related to the morbidity and mortality of liver cancer, which is end-stage liver disease [13], Korea implemented nationwide liver cancer screening in 2003 as part of the National Cancer Screening Program for secondary prevention. Alpha-fetoprotein and ultrasonography tests are provided for men and women aged 40 years or older with chronic HBV or hepatitis C virus (HCV) infection, liver cirrhosis, or chronic liver disease of any cause [30], [31]. However, there is no specific national program for the primary prevention of liver diseases that targets high-risk populations. A previous study conducted by our team suggested that sex, age, awareness of infection status, and monthly household income are associated with liver cancer screening among HBV carriers [32]. Another study also showed that HBV or HCV carriers who knew their infection status sought liver cancer screening more often than carriers unknown infection status during their lifetime [33]. Although awareness of infection status was the most important factor related to undergoing liver cancer screening among HBV carriers, we found that it was not associated with alcohol consumption among HBV carriers, suggesting that we may need a different approach to the primary and secondary prevention of liver diseases in HBV carriers.

This study has several limitations. First, the information related to demographic, socioeconomic, and life style-related factors was self-reported in health surveys, and thus might be subject to information bias and/or recall bias. Although the alcohol consumption measured in household surveys might be underreported [34], it is possible to use such questions in epidemiological studies [35]. In addition, studies have shown that self-reported information regarding risk factors, including alcohol consumption and smoking, was reasonably reproducible, suggesting reasonable reliability [36], [37]. Therefore, although the questions used in KNHANES might not be validated, the bias was expected to be minimal. Second, because hepatitis B surface antibodies and total hepatitis B core antibodies were not assessed in the KNHANES, only current HBV infections could be examined, and we could not be certain whether the individuals with HBsAg were acutely infected or chronic carriers. Third, these results are based on cross-sectional study data, and we did not assess the order of the association between sociodemographic factors and drinking. Despite these limitations, a major strength of the study is that the KNHANES data are representative of the Korean population and, by applying sampling weights in the analysis, the results can be applied to all HBV carriers in Korea. Second, the prevalence of alcohol consumption and its associated factors among HBV carriers may provide opportunities for the primary prevention of liver cirrhosis or HCC in a high-risk population.

HBV infection is endemic in Korea, and the burden of liver diseases related to HBV infection is substantial [14], [15]. Efforts, from primary prevention to tertiary prevention, are necessary across the natural history of disease to decrease the burden related to the morbidity and mortality associated with HBV infection. Previous studies focusing on secondary prevention through liver cancer screening found that the awareness of infection status was an important contributor to undergoing liver cancer screening [32], [33]. However, sociodemographic factors, especially education, were important in terms of primary prevention via healthy lifestyle choices (e.g., abstaining from alcohol consumption). Thus, our findings indicate that efforts to reduce the disease burden caused by HBV infection need to focus on less educated socioeconomic groups to achieve more effective primary prevention. Additionally, other health-related behaviors, such as smoking and obesity, affect alcohol consumption in the population at high risk for HCC. Considering these are other independent and synergistic contributors to the development of HCC in HBV carriers [12], [29], efforts should focus not only on abstinence but also on promoting a generally healthy lifestyle. The difference in alcohol consumption according to educational level among HBV carriers should be considered by health policies or intervention programs that are aimed at HBV carriers and at promoting generally healthy lifestyles.
